# Resource Utilization in Non-Academic Emergency Departments with Advanced Practice Providers

**DOI:** 10.5811/westjem.2019.5.42465

**Published:** 2019-07-01

**Authors:** Ali Aledhaim, Anne Walker, Roumen Vesselinov, Jon Mark Hirshon, Laura Pimentel

**Affiliations:** * University of Maryland School of Medicine, Department of Emergency Medicine, Baltimore, Maryland; † Stanford University School of Medicine, Department of Emergency Medicine, Stanford, California; ‡ University of Maryland School of Medicine, STAR and National Study Center, Baltimore, Maryland

## Abstract

**Introduction:**

Advanced practice providers (APP), including physicians’ assistants and nurse practitioners, have been increasingly incorporated into emergency department (ED) staffing over the past decade. There is scant literature examining resource utilization and the cost benefit of having APPs in the ED. The objectives of this study were to compare resource utilization in EDs that use APPs in their staffing model with those that do not and to estimate costs associated with the utilized resources.

**Methods:**

In this five-year retrospective secondary data analysis of the Emergency Department Benchmarking Alliance (EDBA), we compared resource utilization rates in EDs with and without APPs in non-academic EDs. Primary outcomes were hospital admission and use of computed tomography (CT), radiography, ultrasound, and magnetic resonance imaging (MRI). Costs were estimated using the 2014 physician fee schedule and inpatient payments from the Centers for Medicare and Medicaid Services. We measured outcomes as rates per 100 visits. Data were analyzed using a mixed linear model with repeated measures, adjusted for annual volume, patient acuity, and attending hours. We used the adjusted net difference to project utilization costs between the two groups per 1000 visits.

**Results:**

Of the 1054 EDs included in this study, 79% employed APPs. Relative to EDs without APPs, EDs staffing APPs had higher resource utilization rates (use per 100 visits): 3.0 more admissions (95% confidence interval [CI], 2.0–4.1), 1.7 more CTs (95% CI, 0.2–3.1), 4.5 more radiographs (95% CI, 2.2–6.9), and 1.0 more ultrasound (95% CI, 0.3–1.7) but comparable MRI use 0.1 (95% CI, −0.2–0.3). Projected costs of these differences varied among the resource utilized. Compared to EDs without APPs, EDs with APPs were estimated to have 30.4 more admissions per 1000 visits, which could accrue $414,717 in utilization costs.

**Conclusion:**

EDs staffing APPs were associated with modest increases in resource utilization as measured by admissions and imaging studies.

## INTRODUCTION

Advanced practice providers (APP), including physicians’ assistants (PA) and nurse practitioners (NP), have been increasingly incorporated into emergency department (ED) staffing over the past decade. According to the Emergency Department Benchmarking Alliance (EDBA), ED APP staffing increased from 23% of EDs in 2010 to 62% in 2016.^1^ This rise is in response to increased ED visits, a shortage of emergency medicine (EM)-trained physicians,^2^ and cost constraints. In addition to providing direct ED patient care, APPs serve as transitional providers between the ED and inpatient units as patients wait for beds to become available.^3^ About 10.5% of PAs identify EM as their primary specialty.^4^,^5^ Another 10% specialize in urgent care medicine, according to a North Carolina study.^3^ Proficiency with procedural skills such as laceration repairs and abscess drainage make APPs particularly suitable to ED and urgent care practice.^6^

Data have demonstrated the cost effectiveness of APPs. Their involvement in urgent care settings decreases costs and waiting room time.^7^ APPs on trauma services have been associated with significantly decreased intensive care unit length of stay.^8^ One of the benefits APPs are thought to provide to the overall staffing structure is the ability to free emergency physicians to see higher acuity patients. Phillips and colleagues found that over 90% of APPs see low-acuity patients defined as Emergency Severity Index (ESI) levels 3–5 while 36% of APPs report caring for high acuity (ESI levels 1 and 2) patients.^9^ The variation in ESI levels seen by APPs might be due to differing physician supervision requirements across states that can also influence diagnostic study and admission ordering privileges.

### Importance

There is scant literature examining resource utilization and the cost-benefit of APPs in the ED setting. In a cross-sectional study surveying American College of Emergency Physician council members, NPs were perceived as using significantly more resources than their PA counterparts. In addition, the survey revealed concern for over-testing by all APPs, which abated with experience.^9^ Despite these concerns, APP use in EDs is increasing incrementally over time.^1^

### Goals of This Investigation

The objectives of this study were twofold: 1) to compare resource utilization in EDs that use APPs in their staffing model with those that do not; and 2) to estimate costs associated with the utilized resources.

## METHODS

### Study Design and Setting

We conducted a five-year retrospective secondary data analysis of non-academic EDs that reported data to the EDBA. EDBA is a national, non-profit ED-level database of member organizations that was created to collate and monitor trends of ED performance metrics on an annual basis.^10^,^11^ The EDBA contains data from more than 1100 EDs in the United States, representing over 40 million patient visits.^12^ The data is accessible to member institutions that voluntarily submit their ED demographics and performance metrics to the organization. Member organizations use the data to benchmark performance against similar EDs, identify best practices, conduct research, and collaborate to improve quality. EDBA contains annual ED aggregate data including hospital demographics, annual visit volumes, provider hours, patient acuity, length of stay, hospital admissions, computed tomography (CT), radiographs, ultrasounds, and magnetic resonance imaging (MRI). Definitions of metrics in the report are standardized by the EDBA Board of Directors. Data are blinded at the hospital level but clustered by state. EDBA data is completely free from commercial influence and solely reported for purposes of benchmarking quality. The database has been used for numerous studies published in peer-reviewed journals.^13^–^17^

**Population Health Research Capsule**

What do we already know about this issue?

Emergency departments (ED) with advanced practice providers (APP) have increased from 23% in 2010 to 62% in 2016, but little is known about resource use as measured by admissions and imaging studies.

What was the research question?

Does resource use differ in EDs staffed with attending physicians only vs EDs with APPs in the staffing mix?

What was the major finding of the study?

Non-academic EDs staffing APPs were associated with modest increases in admissions and imaging studies.

How does this improve population health?

Optimizing resources is essential for population health. Better understanding of resource utilization can help ED staffing decisions and health system costs.

We compared resource utilization in EDs that included APPs in their staffing mix with EDs that did not. We then analyzed the cost implications of those differences using the 2014 Centers for Medicare and Medicaid Services (CMS) physician fee schedule and inpatient Diagnoses Related Group (DRG) payments to estimate utilization cost. The term *cost* is used strictly to represent CMS average admission and prospective resource utilization payments. The University of Maryland institutional review board (IRB) exempted this study from IRB review since only de-identified data were examined.

The study included patient encounters that occurred between 2012 and 2016 in 1092 EDs located in 44 states and the District of Columbia. Because data reporting was voluntary, reporting compliance varied among EDs: some reported data for the entire five-year study period while others reported one, two, three, or four years. The use of APP staffing was unchanged for most EDs during the study period as departments either used or did not use APPs in the staffing matrix. This consistency facilitated a two-group comparative panel in which EDs staffing APPs constituted the comparison group while EDs without APPs became the control group.

All EDs reporting data to the EDBA were examined for inclusion in the study. Inclusion criteria were non-teaching general and adult EDs. We excluded EDs classified as “academic” or “teaching” because they have different resource utilization patterns than non-teaching facilities. EM resident physicians have been shown to increase the hospitalization percentage and use of imaging studies relative to attending physicians practicing alone.^18^,^19^ Similar utilization patterns were observed during our data screening leading to exclusion of academic EDs or EDs with residents. We also excluded EDs classified as free-standing, urgent care, or pediatric, as the former two lack admission capabilities while the latter focuses on a pediatric population, which differs in practice from EDs that treat adults. Thirty-eight EDs changed their staffing patterns from one year to the next, adding or eliminating APPs from their staffing matrix. To avoid biasing the findings by the 38 EDs that would appear in both study arms, we excluded these facilities. We used a first-percentile Winsorization approach^20^ for the primary outcomes to identify the highest and lowest outcome outliers, which were flagged and removed from the analysis.

### Outcome Measures

The primary outcome variables used to reflect resource utilization were hospital admission, CT, radiography, ultrasound, and MRI. All outcome variables were reported as rates (number of uses per 100 ED visits). Of the 1054 EDs included in the study, 144 reported zero use of MRI and 11 reported zero use of ultrasound for an entire year. We converted zero values to missing because we could not discern if “zero” meant lack of use, lack of equipment, or lack of accurate reporting. EDBA defines *ED volume* as the total number of annual patient visits; it defines *high acuity* as the percentage of total visits assigned Current Procedural Terminology (CPT) code levels (four, five, or critical care), and defines *provider hours* as the number of staffed hours in an average day. These three potential confounders—annual ED volume, high acuity, and attending hours—that could influence resource utilization were also examined and included in our analytical models.

Cost estimates were obtained from two CMS sources: inpatient admission charge data and Physician Fee Schedule payments. First, we used the inpatient charge data for fiscal year 2014 to compute the average admission cost. The charge file lists average total payments for each Medicare Severity Diagnosis Related Group (MS-DRG). We summed the total payments for all MS-DRGs and averaged them to obtain a grand overall mean for admission cost. The average admission cost was inflation adjusted by 3.5%^21^ to account for rising healthcare costs. Second, we used the CMS Physician Fee Schedule to estimate resource utilization payments. Because these payments vary according to CPT codes, we estimated usage cost by averaging payment for common radiology CPT codes of each imaging study. The supplemental eTable 1 lists the CPT codes that we used with their prospective payments as derived from the CMS Physician Fee Schedule and includes the summed total and average resource payments.

We calculated the estimated resource utilization cost as follows: the utilization difference between EDs with and without APPs was projected per 1000 patients, which was then multiplied by the average resource payment to reflect the estimated resource cost per 1000 patients. For example, if CT use was increased, hypothetically, by 10 scans per 1000 visits in EDs with APPs compared with those without APPS, and if the average payment of one CT is $276, then the total cost for the extra CTs per 1000 patients would be $2,760 (10 × $276). The supplemental eFigure 1 presents a condensed graphical summary of the average cost per single use and the estimated utilization difference.

### Data Analysis

Descriptive statistics are presented as overall means and standard deviations. Mean comparisons using Student’s t-test were employed to compare resource utilization in EDs with and without APPs. The number of EDs reporting data for each variable was also recorded to reflect variations in reporting patterns. We generated adjusted estimates for each outcome using a linear mixed regression with repeated measures. The multivariable model results were presented as means and 95% confidence intervals (CI) adjusted for ED volume, high acuity, and attending hours. All tests were two sided, with a *p*-value <0.05 considered statistically significant. We performed all analyses with SAS 9.4 statistical package (SAS Institute Inc., Cary, North Carolina).

## RESULTS

### Characteristics of Study Population

**Figure 1:**
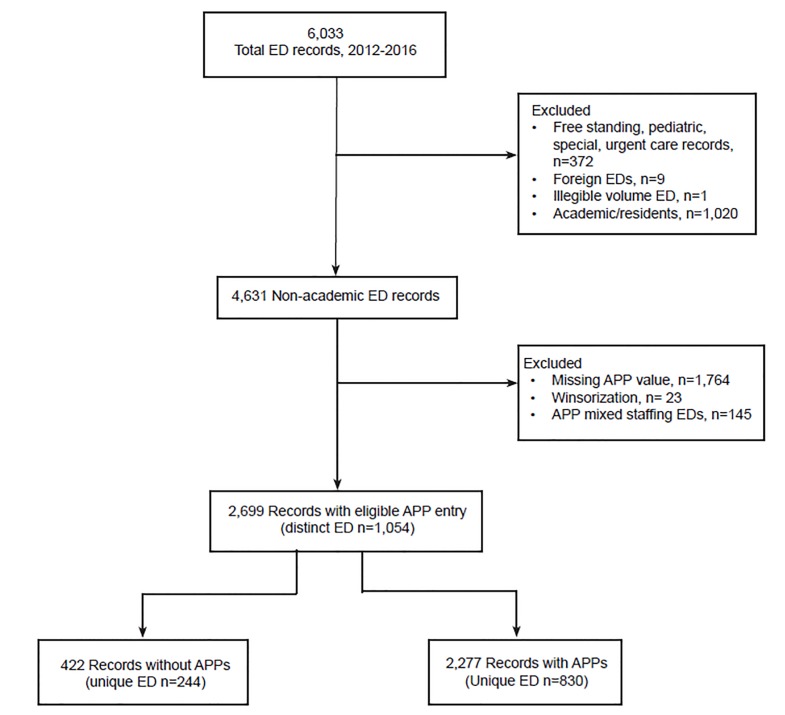
Study population. *ED,* emergency department; *AAP,* advanced practice provider.

The five-year study period contained 6033 total ED records, of which 4631 were for non-academic ED records. After applying our exclusion criteria, the final working sample consisted of 2699 ED records representing 1054 unique EDs (Figure 1).

Of the 1054 distinct EDs, 79% (n=830) had APPs on staff and 21% (n=224) did not. Resource utilization rates by APP status are shown in Table 1. EDs with APPs had higher crude resource utilization rates in all assessed measures compared with EDs without APPs (*p* < 0.05). EDs with APPs also had a higher prevalence of high-acuity visits (66.6% vs 61.3%) and more average attending hours (39.3 vs 28.0 per day) than EDs without APPs.

Adjusted regression estimates comparing resource utilization between EDs with and without APPs are displayed in Table 2. Relative to EDs without APPs, resource utilization increased by 3.04 per 100 visits (95% CI, 2.0–4.1) for admission, by 1.7 per 100 (95% CI, 0.2–3.1) for CT, by 4.5 per 100 (95% CI, 2.2–6.9) for radiography, and by 1.0 per 100 (95% CI, 0.3–1.7) for ultrasound in EDs with APPs. There was no statistical difference in MRI utilization between the two study groups (1.0 vs 0.9 per 100 visits [p=0.58]). The supplemental eTable 2 provides the adjusted coefficient outputs and regression estimates of the controlled covariates for each model.

**Figure 2:**
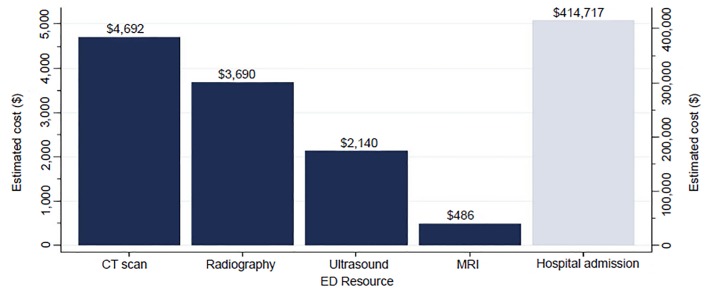
Projected additional costs of emergency departments (ED) with advanced practice providers per 1000 visits, Emergency Department Benchmarking Alliance 2012–2016. *CT,* computed tomography; *MRI,* magnetic resonance imaging.

Figure 2 presents the projected costs associated with increases in resource utilization in EDs with APPs based on 1000 patient visits. The average inflation-adjusted costs were $13,642 for a single hospital admission, $276 for a CT, $82 for a radiograph, $214 for an ultrasound, and $486 for an MRI. EDs with APPs were estimated to have 30.4 more admissions per 1000 patients, which would accrue $414,717 (30.4 × $13,642. While not as substantial, the approximated costs per 1000 for CT, radiography, ultrasound, and MRI were $4692, $3690, $2140, and $486, respectively.

## DISCUSSION

APPs are providing increasing numbers of hours to cover ED shortages in the United States. Using EDBA data, Augustine noted that 39% of hours were worked by PAs or NPs in 2016.^1^ We believe that our study is the first examining the potential impact of this change by comparing resource utilization and cost in the ED setting. Our findings do not conclude that APPs are causing the increase in resource utilizations. We found that EDs staffing APPs were associated with increased resource utilization, as measured by hospital admissions and the use of CT, radiography, and ultrasound studies. Although our study could not directly compare ED attendings to ED APPs due to data limitations, our aggregate analysis demonstrated correlation between APP ED setting and modest increases in utilization. This comparison can be a starting point for future discussions and a call for more robust research on this important topic.

In past studies, other practices outside the ED have shown increased utilization among APPs compared to physicians as well. Studies done in primary care practices demonstrated increased utilization of resources when comparing physician practice with that of APPs. Everett et al. found that patients whose usual primary care provider was an APP had 2.4–3 times the odds of having five or more annual primary care visits (compared with the typical 2–4 visits) if seen primarily by a physician.^22^ In an office-based setting, another study found that new and established patients seen by APPs were significantly more likely to have more imaging studies ordered than those seen by primary care physicians.^23^ Additionally, studies examining quality of care and comparing resource-ordering patterns between APPs and physicians for patients with diabetes or cardiovascular disease found that APPs ordered slightly more tests, imaging studies, and referrals.^24^–^26^

In our study, ultrasound and MRI studies had the smallest adjusted differences, 1 and 0.1 per 100 visits respectively. To evaluate if converting the zero values for 144 MRI and 11 ultrasound records to “missing” biased our model estimates, we included these zero records, re-estimated the models, and compared the findings. We found no sizeable differences between the two models when zero value records were included. Including zero values caused the adjusted differences for ED with APPs to be marginally larger (1.0 vs 1.2 per 100 visits) for ultrasound, and (0.1 vs 0.2 per 100 visits) for MRI, with no change in statistical significance.

Although our findings demonstrate correlation between EDs staffing APPs and increased resource utilization and cost, we cannot assume causation. Our results simply demonstrate that average resource utilization rates increased in EDs with APPs of comparable volume, acuity, and doctor hours relative to EDs without APPs. A valid argument is that resource utilization increases are caused by increases in volume and acuity in EDs staffing APPs. We adjusted for the effect of acuity and volume in our models, and we separately examined if volume or acuity moderated the effect seen on hospital admission. The interaction between acuity and APPs was not statistically significant, which means that EDs with APPs had higher hospital admission rates regardless of acuity levels. On the other hand, patient volume showed a partial moderation effect on hospital admission in that hospital admission differences between EDs with vs those without APPs were larger at EDs with smaller annual volume (<40,000) but the utilization differences were smaller in EDs with larger volume. The direct effect of EDs with APPs was still about three more admissions per 100 visits for EDs with APPs even if the interaction term was adjusted for in the model.

Even with modest differences, the financial impact on the healthcare system can be large. Hospital admission is one of the most prominent factors driving cost. An increase in the rate by 30.4 admissions per 1000 visits in a single hospital could have a projected cost of $414,717. CT, radiography, and ultrasound have projected increases in cost as well, but certainly not as large as hospital admission.

In light of the study limitations, we cannot conclude that APPs are causing the noted increases in resource utilization because multiple factors can influence utilization patterns. Among those factors are clinical experience, medical judgment, and APP scope of practice. Studies examining resource utilization demonstrated that ordering patterns varied among emergency physicians.^27^,^28^ Physicians who ordered more radiography, CTs, ultrasounds, and MRIs were more likely to have higher admission rates than physicians with lower resource utilization rates.^28^ Hence, utilization variations might be due to physician heterogeneity between the two comparative groups.

Scope of practice is another complex factor that can influence resource utilization. While NPs mostly function independently, physician supervision is required for PAs in 43 states.^29^ The level of APPs’ practice is specified by the practice site, which can vary greatly even within the same state. Hence, privileges to order imaging studies or admission independently are diverse among APPs. Some practice sites require co-signature of a physician,^29^ while others do not allow APPs to write admission orders. Our study did not consider the level of attending supervision^19^ in our analysis of resource utilization in EDs staffed with APPs.

This study did not assess quality or patient safety. We do not know if increased utilization represents over-utilization of resources in EDs that staffed APPs or under-utilization in EDs that do not. Further work is needed to delineate and track ordering practices in EDs with and without APPs as well as examine the value of increased testing and hospitalization.

## LIMITATIONS

This study has several limitations. First, it was a retrospective observational study of aggregate data. Control of confounders was limited to aggregate variables available in the data, which included high acuity, annual volume, and average attending hours. The data lacked patient-level (demographic) factors that could influence resource utilization, such as age and comorbidity. ED-specific factors were also limited to those used in the model. Second, we could determine neither the provider who ordered imaging studies or hospitalization, nor the type of APP-physician supervision. Hence, our findings reflect only the overall association between these resources and EDs with APPs. Finally, the CMS cost figures are estimates; actual costs would depend on the specific geographic location and payer mix of the ED patient population.

## CONCLUSION

Within the context of this study’s limitations, we found that EDs staffing APPs are associated with modest increases in resource utilization, as measured by admissions and imaging studies. Studies are needed to track resource utilization prospectively in a more granular ED sample and to incorporate a broader spectrum of diagnoses.

## Supplementary Information



## Figures and Tables

**Table 1:** Descriptive statistics of emergency departments (ED) by advanced practice provider (APP) status (per 100 patient visits), EDBA 2012–2016.^1^

	All EDs (n=1,054)			EDs With APPs (n=830)			EDs without APPs (n=224)			
			
Resource	n	Mean	(SD)	n	Mean	(SD)	n	Mean	(SD)	p value
				
Hospital admission	1021	15.4	±(7.0)	809	16.5	±(6.7)	212	11.3	±(6.6)	<0.01
CT scan	762	20.7	±(7.7)	615	21.4	±(7.7)	147	17.8	±(7.0)	<0.01
Radiography	784	45	±(12.3)	635	46.2	±(12.3)	149	40.1	±(11.5)	<0.01
Ultrasound	450	4.8	±(3.1)	366	5.2	±(3.1)	84	3.1	±(2.5)	<0.01
MRI	610	1.1	±(1.1)	531	1.1	±(1.1)	79	0.8	±(1.0)	0.03
High acuity	938	65.6	±(11.2)	763	66.6	±(10.8)	175	61.3	±(11.6)	<0.01
Attending hours	1053	36.9	±(17.3)	830	39.3	±(17.8)	223	28	±(11.7)	<0.01
Volume	1054	35052	±(21281)	830	40285	±(19908)	224	15661	±(13616)	<0.01

1 Data presented are overall (distinct ED) means and standard deviation (SD); attending hours are average hours on an average day; volume is average per year.

*EDBA,* Emergency Department Benchmarking Alliance; *CT,* computed tomography; *MRI,* magnetic resonance imaging.

**Table 2:** Adjusted resource utilization rates (number of uses per 100 patient visits) for emergency departments (ED) with and without advanced practice providers (APP), EDBA 2012–2016.^1^

Resource	With APPs		Without APPs		Difference		p value
Hospital admission	16.5	(16.1–16.9)	13.5	(12.5–14.4)	3.04	(2.0–4.1)	<0.01
CT scan	20.8	(20.2–21.4)	19.1	(17.8–20.5)	1.7	(0.2–3.1)	0.03
Radiography	45.5	(44.6–46.4)	40.9	(38.8–43.1)	4.5	(2.2–6.9)	<0.01
Ultrasound	4.9	(4.6–5.2)	3.9	(3.2–4.5)	1.0	(0.3–1.7)	0.01
MRI	1.0	(0.9–1.1)	0.9	(0.7–1.1)	0.1	(−0.2–0.3)	0.58

1 Data are means and 95% confidence intervals adjusted for high acuity, volume, and attending hours.

*EDBA,* Emergency Department Benchmarking Alliance; *CT,* computed tomography; *MRI,* magnetic resonance imaging.
