# Supplementary material for: Resource Utilization in Non-Academic Emergency Departments with Advanced Practice Providers
Source: West J Emerg Med. 2019 Jul 1;20(4):541–8. doi: 10.5811/westjem.2019.5.42465 (PMC6625685; doi:10.5811/westjem.2019.5.42465)
Supplement: Supplementary file 1 [file wjem-20-541-s001.docx]

**Resource Utilization in Non-academic Emergency Departments with Advanced Practice Providers**

| **Supplemental eTable.** 2014 Average National Payment of Common Resource CPT Codes as Listed in CMS Physician Fee Schedule.* | | |
| --- | --- | --- |
| COMPUTED TOMOGRAPHY (CT) SCAN | | |
| HCPCS Code | Short Description | OPPS Facility Payment Amount |
| 74150 | Ct abdomen w/o dye | $187.35 |
| 74160 | Ct abdomen w/dye | $313.81 |
| 74178 | Ct abd & pelv 1/> regns | $492.56 |
| 71250 | Ct thorax w/o dye | $178.04 |
| 71260 | Ct thorax w/dye | $312.37 |
| 71270 | Ct thorax w/o & w/dye | $349.99 |
| 74170 | Ct abdomen w/o & w/dye | $351.42 |
| 74177 | Ct abd & pelv w/contrast | $482.89 |
| 70460 | Ct head/brain w/dye | $306.28 |
| 73200 | Ct upper extremity w/o dye | $178.04 |
| 73201 | Ct upper extremity w/dye | $308.43 |
| 73700 | Ct lower extremity w/o dye | $178.04 |
| 73701 | Ct lower extremity w/dye | $308.43 |
| 70450 | Ct head/brain w/o dye | $169.80 |
| 70470 | Ct head/brain w/o & w/dye | $345.33 |
| 70487 | Ct maxillofacial w/dye | $315.24 |
| 70491 | Ct soft tissue neck w/dye | $319.18 |
| 70480 | Ct orbit/ear/fossa w/o dye | $191.65 |
| 70481 | Ct orbit/ear/fossa w/dye | $319.54 |
| 70482 | Ct orbit/ear/fossa w/o&w/dye | $354.29 |
| 70490 | Ct soft tissue neck w/o dye | $191.65 |
| 72193 | Ct pelvis w/dye | $308.43 |
| 70486 | Ct maxillofacial w/o dye | $184.49 |
| 72126 | Ct neck spine w/dye | $311.30 |
| 72192 | Ct pelvis w/o dye | $181.62 |
| 72194 | Ct pelvis w/o & w/dye | $342.11 |
| 76380 | Cat scan follow-up study | $128.96 |
| 72125 | Ct neck spine w/o dye | $180.55 |
| 72128 | Ct chest spine w/o dye | $177.32 |
| 72129 | Ct chest spine w/dye | $311.30 |
| 72131 | Ct lumbar spine w/o dye | $177.32 |
| 72132 | Ct lumbar spine w/dye | $311.30 |
| 74176 | Ct abd & pelvis w/o contrast | $330.64 |
| Total Payment |  | $9,099.67 |
| Average CT Scan Payment | | $275.75 |
| MAGNETIC RESONANCE IMAGING (MRI) | | |
| 70551 | Mri brain stem w/o dye | $370.05 |
| 70553 | Mri brain stem w/o & w/dye | $609.70 |
| 74181 | Mri abdomen w/o dye | $368.97 |
| 74183 | Mri abdomen w/o & w/dye | $607.55 |
| 77059 | Mri both breasts | $576.03 |
| 71550 | Mri chest w/o dye | $368.97 |
| 71552 | Mri chest w/o & w/dye | $607.55 |
| 73718 | Mri lower extremity w/o dye | $363.60 |
| 73720 | Mri lwr extremity w/o&w/dye | $601.82 |
| 73721 | Mri jnt of lwr extre w/o dye | $365.03 |
| 73218 | Mri upper extremity w/o dye | $363.60 |
| 73220 | Mri uppr extremity w/o&w/dye | $602.18 |
| 73221 | Mri joint upr extrem w/o dye | $365.03 |
| 73223 | Mri joint upr extr w/o&w/dye | $602.18 |
| 73723 | Mri joint lwr extr w/o&w/dye | $602.18 |
| 70540 | Mri orbit/face/neck w/o dye | $363.60 |
| 70543 | Mri orbt/fac/nck w/o &w/dye | $601.82 |
| 72195 | Mri pelvis w/o dye | $370.05 |
| 72197 | Mri pelvis w/o & w/dye | $607.55 |
| 72141 | Mri neck spine w/o dye | $370.77 |
| 72146 | Mri chest spine w/o dye | $370.77 |
| 72148 | Mri lumbar spine w/o dye | $371.12 |
| 72156 | Mri neck spine w/o & w/dye | $609.70 |
| 72158 | Mri lumbar spine w/o & w/dye | $610.42 |
| 70336 | Magnetic image jaw joint | $369.69 |
| 72157 | Mri chest spine w/o & w/dye | $609.70 |
| Total Payment |  | $12,629.63 |
| Average MRI Payment | | $485.76 |
| RADIOGRAPHY (X-ray) | | |
| 71010 | Chest x-ray 1 view frontal | $66.63 |
| 73050 | X-ray exam of shoulders | $68.78 |
| 73610 | X-ray exam of ankle | $66.27 |
| 74000 | X-ray exam of abdomen | $66.63 |
| 74020 | X-ray exam of abdomen | $70.93 |
| 71020 | Chest x-ray 2vw frontal&latl | $68.42 |
| 71022 | Chest x-ray frnt lat oblique | $74.15 |
| 71035 | Chest x-ray special views | $66.63 |
| 73000 | X-ray exam of collar bone | $65.91 |
| 77072 | X-rays for bone age | $66.99 |
| 70030 | X-ray eye for foreign body | $65.91 |
| 70150 | X-ray exam of facial bones | $104.24 |
| 73080 | X-ray exam of elbow | $66.27 |
| 73140 | X-ray exam of finger(s) | $64.48 |
| 73090 | X-ray exam of forearm | $65.91 |
| 73130 | X-ray exam of hand | $66.27 |
| 73630 | X-ray exam of foot | $65.91 |
| 73650 | X-ray exam of heel | $65.56 |
| 70110 | X-ray exam of jaw 4/> views | $103.53 |
| 73060 | X-ray exam of humerus | $66.27 |
| 73562 | X-ray exam of knee 3 | $67.71 |
| 74400 | Contrst x-ray urinary tract | $282.64 |
| 70160 | X-ray exam of nasal bones | $66.27 |
| 70200 | X-ray exam of eye sockets | $71.65 |
| 71101 | X-ray exam unilat ribs/chest | $104.24 |
| 72170 | X-ray exam of pelvis | $66.99 |
| 77074 | X-rays bone survey limited | $114.27 |
| 71111 | X-ray exam ribs/chest4/> vws | $107.11 |
| 71130 | X-ray strenoclavic jt 3/>vws | $68.78 |
| 73010 | X-ray exam of shoulder blade | $67.71 |
| 73030 | X-ray exam of shoulder | $67.71 |
| 70220 | X-ray exam of sinuses | $70.21 |
| 70260 | X-ray exam of skull | $108.54 |
| 70360 | X-ray exam of neck | $65.91 |
| 72040 | X-ray exam neck spine 2-3 vw | $69.50 |
| 72202 | X-ray exam si joints 3/> vws | $66.99 |
| 72050 | X-ray exam neck spine 4/5vws | $107.47 |
| 72052 | X-ray exam neck spine 6/>vws | $109.98 |
| 72100 | X-ray exam l-s spine 2/3 vws | $69.50 |
| 72110 | X-ray exam l-2 spine 4/>vws | $107.47 |
| 72114 | X-ray exam l-s spine bending | $108.54 |
| 71120 | X-ray exam breastbone 2/>vws | $68.06 |
| 72072 | X-ray exam thorac spine 3vws | $101.74 |
| 72090 | X-ray exam scloiosis erect | $73.44 |
| 72120 | X-ray bend only l-s spine | $103.17 |
| 73590 | X-ray exam of lower leg | $66.27 |
| 70330 | X-ray exam of jaw joints | $70.57 |
| 73110 | X-ray exam of wrist | $66.27 |
| 73660 | X-ray exam of toe(s) | $64.12 |
| Total Payment |  | $3,998.52 |
| Average Radiography Payment | | $81.60 |
| ULTRASOUND (US) | | |
| 76700 | Us exam abdom complete | $175.53 |
| 76705 | Echo exam of abdomen | $164.43 |
| 76857 | Us exam pelvic limited | $109.62 |
| 93880 | Extracranial bilat study | $221.38 |
| 93306 | Tte w/doppler complete | $491.85 |
| 93925 | Lower extremity study | $231.42 |
| 93926 | Lower extremity study | $160.13 |
| 93970 | Extremity study | $226.40 |
| 76536 | Us exam of head and neck | $162.99 |
| 76805 | Ob us >/= 14 wks sngl fetus | $184.49 |
| 76881 | Us xtr non-vasc complete | $166.93 |
| 93971 | Extremity study | $157.26 |
| 76604 | Us exam chest | $161.92 |
| 76770 | Us exam abdo back wall comp | $171.95 |
| 76801 | Ob us < 14 wks single fetus | $184.49 |
| 76817 | Transvaginal us obstetric | $173.02 |
| 76856 | Us exam pelvic complete | $169.08 |
| 76775 | Us exam abdo back wall lim | $164.07 |
| 76830 | Transvaginal us non-ob | $169.44 |
| 76870 | Us exam scrotum | $166.93 |
| 93351 | Stress tte complete | $680.63 |
| Total Payment |  | $4,493.96 |
| Average Ultrasound Payment | | $214.00 |

*CPT,* Current Procedural Terminology; *CMS*, Centers for Medicare & Medicaid Services; *HCPCS,* Healthcare Common Procedure Coding System; *OPPS,* Outpatient Prospective Payment System; *abd,* abdominal; *pelv,* pelvic; *w/o,* without; *w,* with; *upr extre*, upper extremities; *orbt*, orbital; *fac,* face; *nck*, neck; *ob,* obstetrics; *tte,* transthoracic and transesophogeal; *xtr,* extremities; *vasc,* vascular;

*Global modifier was applied in fee search


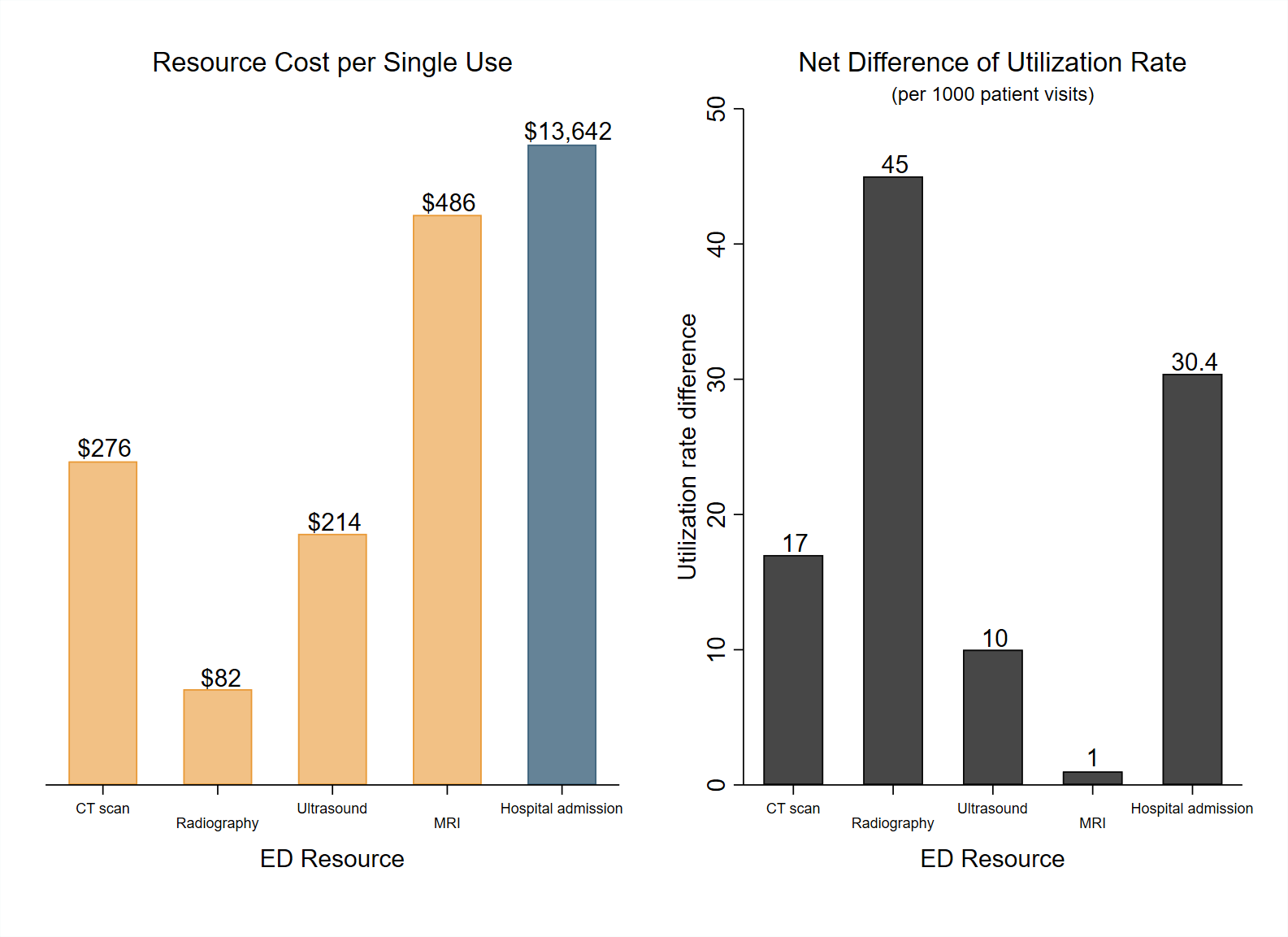


**eFigure 1.** Summary of the average cost per single use and the estimated utilization difference of emergency departments (ED) staffing advanced practice providers.

| eTable 2. Adjusted regression estimates for each assessed model (difference per 100 patient visits), Emergency Department Benchmarking Alliance 2012-2016^1^ | | | | | |
| --- | --- | --- | --- | --- | --- |
| Models | Elements | Estimated difference | 95% Confidence Interval | | *p*-value |
| Model 1: Hospital admission | ED with APP | 3.04 | 2.0 | 4.08 | <0.001 |
|  | Acuity | 0.09 | 0.07 | 0.1 | <0.001 |
|  | ED volume | 2.30E-05 | 4.21E-06 | 4.20E-05 | 0.016 |
|  | Attending hours | 0.08 | 0.06 | 0.09 | <0.001 |
| Model 2: CT Scan | ED with APP | 1.67 | 0.19 | 3.14 | 0.027 |
|  | Acuity | 0.13 | 0.1 | 0.16 | <0.001 |
|  | ED volume | 8.10E-05 | 4.70E-05 | 1.15E-04 | <0.001 |
|  | Attending hours | -0.02 | -0.06 | 0.01 | 0.21 |
| Model 3: Radiography | ED with APP | 4.54 | 2.21 | 6.88 | <0.001 |
|  | Acuity | 0.03 | -0.03 | 0.08 | 0.29 |
|  | ED volume | 3.20E-05 | -2.40E-05 | 8.90E-05 | 0.257 |
|  | Attending hours | 0.11 | 0.05 | 0.17 | 0.001 |
| Model 4: Ultrasound | ED with APP | 1.02 | 0.29 | 1.74 | 0.006 |
|  | Acuity | 0.04 | 0.02 | 0.05 | <0.001 |
|  | ED volume | 2.50E-05 | 8.64E-06 | 4.30E-05 | 0.003 |
|  | Attending hours | 0.01 | -0.002 | 0.03 | 0.101 |
| Model 5: MRI | ED with APP | 0.07 | -0.17 | 0.31 | 0.581 |
|  | Acuity | 0.01 | 0.007 | 0.014 | <0.001 |
|  | ED volume | 7.40E-06 | 3.28E-06 | 1.15E-05 | <0.001 |
|  | Attending hours | 9.10E-04 | -2.70E-03 | 4.48E-03 | 0.617 |

*ED,* emergency department; *APPs,* advanced practice providers; *CT,* computed tomography; *MRI,* magnetic resonance imaging

^1^Data are average differences and 95% CI of EDs with APPs compared to EDs without APPs, adjusted for high acuity, volume, and attending hours.
